# Boosted Antioxidant Effect Using a Combinatory Approach with Essential Oils from *Origanum compactum*, *Origanum majorana*, *Thymus serpyllum, Mentha spicata, Myrtus communis*, and *Artemisia herba-alba*: Mixture Design Optimization

**DOI:** 10.3390/plants10122817

**Published:** 2021-12-20

**Authors:** Wessal Ouedrhiri, Hamza Mechchate, Sandrine Moja, Ramzi A. Mothana, Omar M. Noman, Andriy Grafov, Hassane Greche

**Affiliations:** 1 Laboratory of Inorganic Chemistry, Department of Chemistry, University of Helsinki, P.O. Box 55, FI-00014 Helsinki, Finland; Andriy.grafov@helsinki.fi; 2 Université de Lyon, UJM-Saint-Etienne, CNRS, BVpam, FRE3727, F-42023 Saint-Etienne, France; Sandrine.moja@univ-st-etienne.fr; 3 Department of Pharmacognosy, College of Pharmacy, King Saud University, Riyadh 11451, Saudi Arabia; Rmothana@ksu.edu.sa (R.A.M.); onoman@ksu.edu.sa (O.M.N.); 4 National Agency of Medicinal and Aromatic Plants, University Sidi Mohamed Ben Abdellah, Taounate BP 159, Morocco; hgreche@yahoo.fr

**Keywords:** antioxidant, essential oil, mixture design, DPPH, optimal combination, medicinal plants

## Abstract

Several studies have demonstrated the possible synergistic effect as an effective strategy to boost the bioactivity of essential oils. Using this framework, this study was conducted to effectively establish the ideal combination of six essential oils from different plants (*Origanum compactum*, *Origanum majorana*, *Thymus serpyllum*, *Mentha spicata*, *Myrtus communis*, and *Artemisia herba-alba*) that would express the best antioxidant activity. Each mixture was optimized using a mixture design approach to generate the most effective blend. The 2,2-diphenyl-1-picrylhydrazyl radical scavenging method was used as a reference method to assess the antioxidant activity. Each essential oil’s composition was identified using the GC/MS method. The single essential oil activities demonstrated variable antioxidant effects, and following the mixture design approach, the optimal antioxidant blend was revealed, as two mixtures demonstrated the best antiradical activity with 79.46% obtained with the mixture of *O. majorana* (28%) and *M. spicata* (71%) and 78.8% obtained with the mixture *O. compactum* (64%), *O. majorana* (13%), and *T. serpyllum* (21%). This study proposes a practical way to elaborate mixtures in the search for a boosting effect that can be oriented for the food or pharmaceutical industry.

## 1. Introduction

Free radicals are the main products of oxidation. They represent reactive chemical species that possess an unpaired electron on the outer orbit and are both stable in their shape [1,2]. The oxidation reaction in the body and in food has been widely recognized. It can cause the degradation of various oxygen substrates, polyunsaturated fatty acids, phospholipids, cholesterol, lipids, and DNA. Oxidation control in various fields is based on the use of antioxidants, which are compounds that can slow or retard the oxidation of an oxidizable material, even if they are used in a very small amount (<1%, usually 1 to 1000 mg/L) in relation to the amount of material they need to protect [3]. According to their mechanism of action, antioxidants are classified into two categories of preventatives acting through three different mechanisms: the decrease of oxygen concentration and the prevention of initiation chains by (i) trapping initiator radicals (ii) blocking the metal ions that are responsible for hydroxyls, ferryl Fe^2+/^Fe^3+^/O_2_ generation, and/or (iii) breaking down lipid peroxides with peroxyl or alkoxyl radicals. However, primers react through two mechanisms: the decomposition of peroxides by their conversion to non-radical products; or by breaking the reaction chains, trapping the intermediate radicals, and preventing the abstraction of continuous hydrogen [4]. Due to increased concerns regarding the safety of chemical and synthetic preservatives, alternative mechanisms based on the use of natural compounds have been increasingly tested over the last years [5].Essential oils are used in the food industry and in cosmetics due to their preservative effect because of the presence of antioxidant constituents and due to the flavoring and dyeing properties of some medicinal plants [6]. Recently, the interest in essential oils and their application in preservation has been amplified [7] by an increasingly negative perception of synthetic preservatives by consumers. In fact, synthetic antioxidants, such as butylated hydroxyanisole (BHA) and butylated hydroxytoluene (BHT), are frequently used to prevent or delay oxidation. However, BHA and BHT are known to cause human health risks [8]. The antioxidant properties of essential oils and their constituents have been widely documented. Pioneering work has also elucidated some mechanisms of a few oil constituents, but details are still lacking [3]. This property could not always be obtained in several industries by only adding the essential oil in high quantities due to the interaction of the essential oil with the excipient. Consequently, several studies have confirmed that synergies might be produced using several essential oils as a solution. However, little is known about the interactions that cause synergistic, additive, or antagonistic effects. Such knowledge could contribute to the design of new and more potent antioxidant mixtures and to understanding the interaction between the constituents of crude essential oils. All of this requires an overview of current knowledge about the antioxidant properties and antioxidant mode of action of essential oils and their constituents, as well as identifying possible avenues of research that may facilitate the use of EO as preservatives. Among the methods employed for studying the antioxidant interactions of EOs, we cite the most known: checkerboard assay, where the interaction is identified using a simple linear regression [9]. In this research paper, we use a new conception of the mixture to evaluate the antioxidant effect produced by the interaction of different EOs, based on multiple linear regression. This approach can guide the use of recipes of EOs by indicating the sufficient concentrations leading to synergistic interactions. The antioxidant activity of six EO mixtures was tested in this study. The first mixture was prepared with *Origanum compactum*, *Origanum majorana*, and *Thymus serpyllum* essential oils. They allowed the combination of three major terpene families: phenols (carvacrol, thymol), alcohols (*trans*-thujanol, terpinene-4-ol), and hydrocarbons (*p*-cymene, γ-terpinene). The second blend was formulated with *O. majorana*, *Mentha spicata*, and *T. serpyllum*, essential oils widely used in cooking. This mixture allowed the interaction of three major families, alcohols ((-)terpinene-4-ol, trans-Thujanol), a ketone (carvone), and hydrocarbons (*p*-cymene and γ-terpinene). The third mixture was formed by *Myrtus communis*, *Artemisia herba-alba*, and *T. serpyllum* EOs. It allowed assembling mainly ether, ketone, and monoterpene hydrocarbons. 

## 2. Results and Discussion 

### 2.1. Essential Oil Yield and Chemical Composition 

The hydrodistillation of the aerial part of *O. compactum* EO produced a yield of 1%. Analysis of its chemical composition named twenty-four compounds representing a cumulative area that corresponded to 99.65% of the cumulative areas of all constituents. The oxygenated monoterpenes represented 65.87% of the identified compounds, the hydrocarbon monoterpenes represented 32.1%, while the hydrocarbon sesquiterpenes did not exceed 1.44%, represented by caryophyllene (GC-MS results are presented in the supplementary file). A predominance of carvacrol (47.80%), γ-terpinene (17.25%), and thymol (15.74%) was observed. Concerning the hydrodistillation of the aerial part of *O. majorana*, a yield of 1.2% was obtained. The CG/MS analysis revealed twenty-one compounds accounting for 95.72% of total accumulated air. The major components of marjoram EO were: (-)-terpine-4-ol (29.10%), *trans*-4-thujanol (24.57%), and *p*-cymene (12.64). The terpene derivatives encompassed 93.42% of monoterpenes and 2.3% of sesquiterpenes, where the hydrocarbon monoterpenes represented 16.87%, and oxygenated 76.55%, while the hydrocarbon sesquiterpenes were represented by caryophyllene at 0.4%, against 1.9% for oxygenated sesquiterpenes. The hydrodistillation of *T. serpyllum* EO revealed a yield of 0.9%. Analysis of the chemical composition identified thirty-six compounds representing 99.61%. Among the identified terpene compounds, 96.08% of monoterpenes were found against only 3.53% of sesquiterpenes. The monoterpene fraction was represented by the hydrocarbon monoterpenes representing the majority class with a rate of 67.21%, and by the oxygenated monoterpenes representing 28.87%. However, the sesquiterpenes were only represented by those with a hydrocarbon content of 3.53%. Three monoterpenes, *p*-cymene (36.15%), γ-terpinene (18.31%), and thymol (17.29%) were identified as major compounds. The *M. spicata* EO yield was of 0.7%. Fifty-five compounds were identified in the CG/MS analysis, forming 96.34% of the total oil, with a remarkable abundance of monoterpenes accounting for 31.53% of those hydrocarbons and 43.68% of those oxygenated. While the sesquiterpenes represented only 10.22% of those hydrocarbons, and 0.3% of those oxygenated. Four major compounds were identified, carvone (26%), 1,8-cineole (15.2%), β-mycene (12.5%), and limonene (10.29%). Hydrodistillation of *M. communis* leaves gave a yield of 0.5%.Twenty-seven compounds were identified during EO leaf analysis of *M. communis* leaves, constituting 90.3% of the total oil. The chemical composition of this oil is marked by the abundance of terpene derivatives representing 36.15%, the oxygenated monoterpenes 35.16%, and the hydrocarbon monoterpenes which represented 17.62%. However, sesquiterpenes were present in trace form at 1.37%. The main EO compounds in *M. communis* leaves were myrtenyl acetate (33.67%), 1,8-cineole (19.77%), and limonene (8.96%). Hydrodistillation of the aerial part of *A. herba-alba* recorded a yield of 1%. The analysis of the EO identified eighteen compounds constituting 94.58% of the total amount of oil. The terpene composition was marked by the strong presence of oxygenated monoterpenes forming 88.7% of the oil and represented mainly by the piperitone chemotype forming 85.68% of the total oil, followed by the oxygenated sesquiterpenes representing 3.38%. However, monoterpene and sesquiterpene hydrocarbons represented only 1.99% and 0.15%, respectively. 

### 2.2. Antioxidant Screening 

Table 1 collates the IC50 obtained for the studied EOs, as well as that of the BHT.

*T. serpyllum* and *M. spicata* recorded the lowest IC50s, which reflects their strong antioxidant effects that exceeded that of BHT. Generally, all the oils showed a strong ability to trap the DPPH radical.

EOs are complex mixtures of compounds belonging to different chemical families, and several factors are responsible of their varying antioxidant activities. The authors have often attributed a strong antioxidant activity to the presence of relatively high amounts of phenolic compounds, such as thymol and carvacrol [4], whose ability to trap the DPPH radical has already been demonstrated by Aazza et al. [10]. Thus, alcohols such as citronellol and geraniol have also shown a high antioxidant capacity [11,12], so have others, such as α-pinene, *p*-cymene, linalool, linalyl acetate, 1,8-cineole, limonene, borneol, and δ-3-Carene.

Therefore, it can be said that the overall performance as an antioxidant is, in fact, the result of the complex interaction between the components, producing a synergistic or antagonistic behavior. Kulisic et al. [13], showed some of the different antioxidant behaviors of EOs. In fact, the *Origanum vulgare* L. EO containing 67% of thymol plus carvacrol and ~14% of γ-terpinene, offers an example of synergy between the components of the oils. By contrast, its isolated fraction of hydrocarbons offered no antioxidant effect, and the oxygenated fraction (containing about 94% thymol + carvacrol) was no different than isolated carvacrol or thymol alone. On the other hand, *Satureja montana* L. EO, containing ~50% of thymol and carvacrol, and only 6% of γ-terpinene, gives the same effect as its oxygenated fraction (~70% of carvacrol + thymol), slightly less effective than pure carvacrol or thymol, while the hydrocarbon fraction provided a negligible effect. In other words, the antioxidant behavior was simply that expected from the contents of the most effective components. The oils of the two species of thymus (*Thymus vulgaris* L. and *Thymus serpyllum* L.), containing about 80% thymol + carvacrol and ~5.5% of γ-terpinene each, had a somewhat intermediate behavior. By contrast, *Satureja cuneifolia* L., containing only 13% phenols, no γ-terpinene, and an abundance of unsaturated terpenoids such as linalool, was influenced by the intense pro-oxidative effect of its hydrocarbon fraction neutralizing the antioxidant behavior of the oxygenated fraction. 

### 2.3. Formulations by Mixture Design 

#### 2.3.1. Formulations’ Antioxidant Activity 

Three mixtures were carried out. The first mixture, M1, combined the EOs of *O. compactum*, *O. majorana*, and *T. serpyllum*; the second mixture, M2, combined the Eos of *O. majorana*, *T. serpyllum*, and *M. spicata*, the third mixture, M3, associated the *A. herba-alba*, *M. communis*, and *T. serpyllum* EOs (Table 2).

The antioxidant activity obtained from every formulation is presented in Table 3; values of M1 varied between 79.55% for the half–half formulation of *O. compactum* and *T. serpyllum,* and 47.3% with *O. majorana* EO. For M2, values balanced between 79.55 of *O. majorana* and *M. spicata* (50%/50%) and 47.3 of *O. majorana* EO alone. Mixture M3 values varied between 78.5% of *M. communis* oil alone and 8.15% with the half–half *M. communis* and *A. herba-alba* mixture. 

#### 2.3.2. Statistical Validation of the Postulated Model 

Experimental responses (Table 3) were submitted to mathematical statistical analysis applying the principle of linear regression to predict the equations describing antioxidant activity variations in terms of mixtures. Table 4 shows that the effect of linear regression is significant, since the probability of the significance of risk p-value is less than 0.05%. The coefficient of determination R^2^ of 0.96, 0.97, 0.99 of the mixtures M1, M2, and M3, respectively, have satisfactory values for describing the shape of the curve representing the experimental values as a function of the values predicted by the mathematical model. Concerning the calculated coefficients, a negative sign of a coefficient in the adjusted model indicates the ability of its associated factor to decrease the response, while a positive sign shows the ability of a factor to increase the response variable. In this study, the goal was to increase the combined antioxidant effect, that is, to increase the response variables (AA values). So, positive sign of a coefficient shows the ability of its associated factor to increase antioxidant activity.

The mathematical model (linear regression) approaching 1 means that experimental value and estimated value are correlated; this was the case with different studies using the mixture design experiment in different applications such as preparing a formulation of EOs with the most potent antibacterial activity [14] and preparing a formulation containing bioactive molecules to treat diabetes and hyperglycemia [15,16].

#### 2.3.3. Test-Point

To finalize the validity tests of the selected model, we used the point-test tool. Thus, we performed a formulation whose result corresponded to the desired response. The coordinates of the point-test chosen were: X1 = 0.16667, X2 = 0.16667, X3 = 0.66667. They allowed to obtain a radical inhibition of 67.5%, 70.12%, and 52% (for mixtures M1, M2, and M3, respectively); values which are very close to that obtained by the mathematical model equal to 70.13%, 72.47%, and 49.43%, respectively.

#### 2.3.4. Effect of Mixture Components and Their Interactions on the Responses

**Figure 1:**
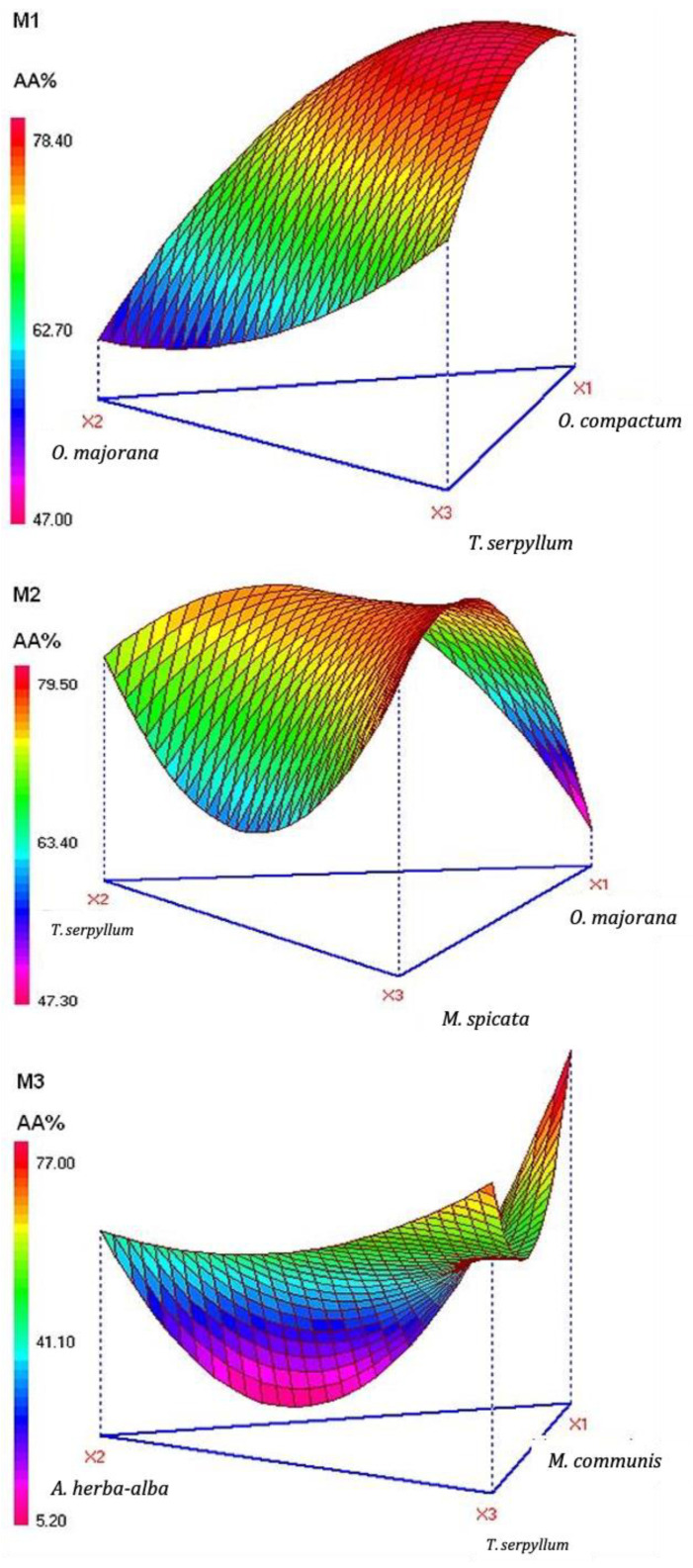
Three dimensional surface plots for the effect of different combinations of studied essential oils mixture, M1, M2, and M3.

The description of the variations in antioxidant activity is represented as a 3D figure (Figure 1).

The magnitudes of the interactions between EOs reveal the interaction produced and explain antioxidant activity variations. In mixture M1, a significant synergism effect between oregano and marjoram (*p* = 0.0006) and oregano and thyme (*p* = 0.0279) EOs was found, while, any ternary antioxidant effect was detected. Regarding mixture M2, three significant binary interactions were observed, where their positive magnitudes expose the synergistic antioxidant effect. However, all of the binary interactions produced an antagonistic antioxidant effect in mixture M3, except the ternary interaction that produced a high synergistic antioxidant activity.

Following significance of each coefficient (Table 5, Table 6 and Table 7), the Equations (1)–(3) obtained for mixtures M1, M2, and M3, respectively, are:

(1) y=75.04Oc+49.81Om+68.02Ts+42.25OcOm+20.78OcTs

(2) y=49.81Om+68.02Ts+74.90Ms+56.29OmTs+57.48OmMs−63.69TsMs

(3) y=76.95Mc+45.87Aha+68.02Ts−219.02McAha−114.80McTs−38.02AhaTs+359McAhaTs

The antioxidant activity variations were significant in the three mixtures studied. In fact, the combination of *T. serpyllum* EO in three different mixtures, and combination of *O. majorana* in two others, showed different interactions outcomes. Besides, the interaction between EOs in the case of antioxidant activity has already been observed by other authors, and a phenomenon of synergy or antagonism has been also demonstrated. In fact, binary combinations of the EOs of *Brassica nigra*, *Cuminum cyminum*, and *Coriandrum sativum* have shown synergistic and additive interactions for the reduction of the free radical DPPH [17]. Thus, Bag et al., tested a combination of *Angelica archangelica* EO, phenyl ethyl alcohol, and α-terpineol, and a potential antioxidant effect was obtained with an IC_50_ value of 3.89 μL/mL [9]. 

The interaction between synthetic antioxidants was also evaluated by Yi et al. [18], who illustrated the synergistic effect existing between tocopherol and ascorbic acid. Thus, Kurechi and Kato [19] proved the synergistic effect between butylhydroxyanisole BHA and butylhydroxytoluene BHT on the free radical DPPH. A ternary mixture of BHA, TBHQ terbutylhydroquinone, and BHT was subjected to the mixture design method to evaluate the oxidation stability of B100 biodiesel. This study has highlighted the synergistic interaction produced by the binary mixture of BHA and TBHQ which is the most efficient mixture for the protection of B100 biodiesel from oxidation [20]. 

In M1, the interactions between *O. compactum* oil and that of *O. majorana*, and their interaction with *T. serpyllum* oil were significant, and positively influenced the antioxidant activity of this mixture. In M2, the interaction of marjoram oil with that of wild thyme and mint was significant and positively influenced its antioxidant effect. However, the interaction of wild thyme oil with mint was significant, but it negatively influenced the antioxidant effect. On the other hand, in M3, all the binary interactions between myrtle, mugwort, and wild thyme, negatively influenced the antioxidant effect of this mixture, contrary to the ternary effect.

These interactions are mainly due to the chemical profile of each EO. Several mechanisms have been proposed to explain this phenomenon. Indeed, the interaction between carvacrol and thymol showed synergistic antioxidant effect when it was tested by the Llana-Ruiz-Cabello team [21]. Although they do not employ the same mechanism of action as was shown by Yanishlieva et al. [22]. γ-terpinene, having a cyclohexadiene structure, acts as an antioxidant thanks to its auto-oxidation [3]. Research also showed its synergistic effect when mixed with rutin which is a polyphenol [23]. Monoterpene *p*-cymene, considered a substantial antioxidant, has already shown a synergistic antioxidant effect with thymol and/or carvacrol, while demonstrating an antagonistic effect with thymoquinone. However, this latter mixture produces a synergistic antioxidant effect when added to thymol and/or carvacrol [24].

Previous studies have also been involved in explaining the synergy between EOs. In fact, they have reported that unsaturated carbon–carbon double bonds (C = C) are able to inhibit radical reactions by attracting simple electrons from free radicals to form a stable electron cloud system [25]. Others classify antioxidants as primary ones that disrupt the oxidative chain reaction of free radicals by donating electrons or hydrogen atoms to the phenolic hydroxyl groups and, therefore, stabilize lipid free radicals, inhibit or slow down the initiation phase, and disrupt the propagation stage of auto-oxidation, and secondary ones that deactivate singlet oxygen, chelated metal ions, absorb ultraviolet radiation, eliminate oxygen, and help regenerate antioxidants primary. They axiomatize the antioxidant synergy in the combination of primary antioxidants with secondary ones [26].

#### 2.3.5. Formulation Optimization

**Figure 2:**
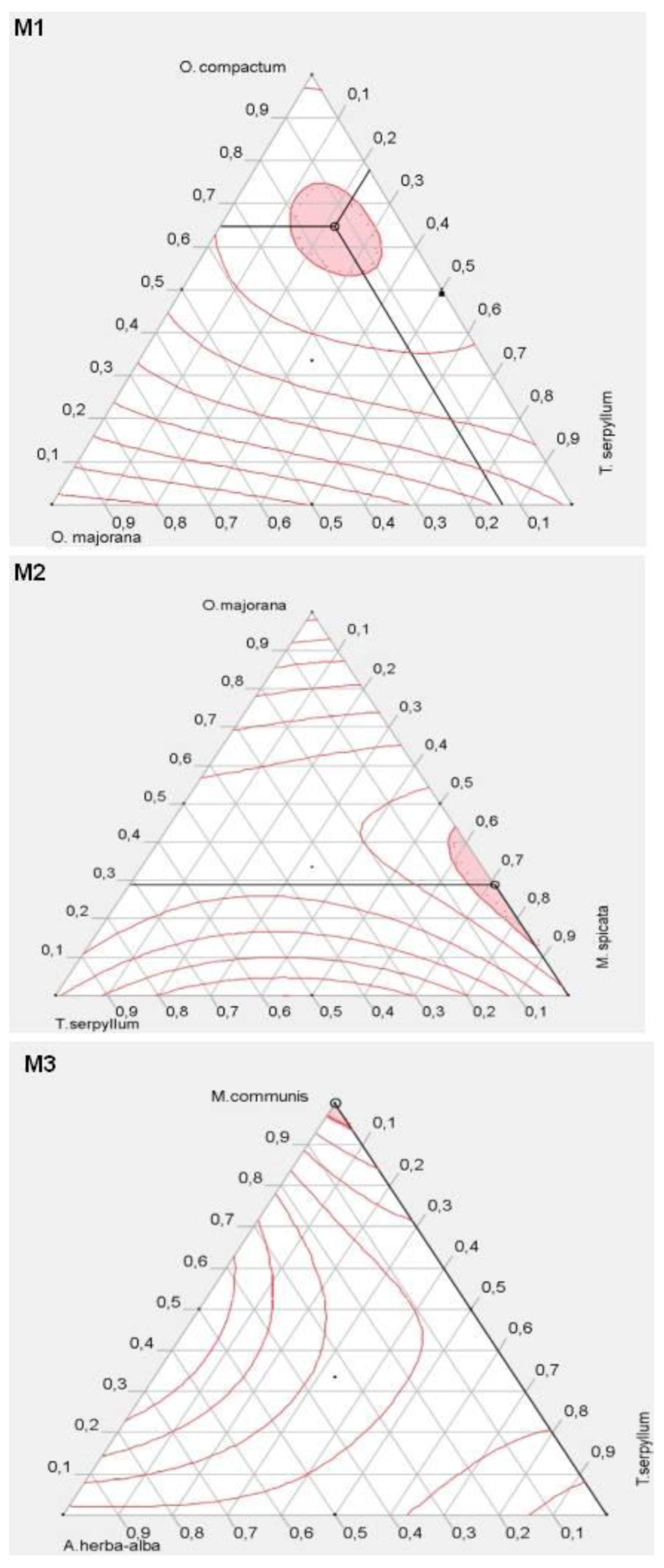
Optimal design regions for the antioxidant effect of the mixtures M1, M2, and M3.

For antioxidant activity, the goal in this study is always to maximize the response. The pink zone in Figure 2. (M1) illustrates the formulations that are giving antioxidant activity that exceeds 78%. The desirability study shows that the optimal antioxidant activity to be obtained is 78.8% with a mixture of 64% of *O. compactum*, 13% of *O. majorana*, and 21% *T. serpyllum*. The mixture profiler in Figure 2 (M2) illustrates the pink zone grouping the mixtures giving an antioxidant activity greater than 78%. The desirability study showed that the optimal antioxidant activity to be achieved is of 79.46% with a mixture of 28% of *O. majorana* oil and 71% of *M. spicata*. The profiler of the mixture in Figure 2. (M3) illustrates the zone that makes possible to obtain an antioxidant activity greater than 70%. No mixture is qualified to give a better antioxidant effect than *M. communis* oil alone, where the activity reaches 76.95%.

The selected plants and their EOs also present a good safety profile that encourages their further application in diverse sectors. Mezzoug et al. confirmed that *O. compactum* does not show any mutagenic activity using the somatic mutation and recombination test in *D. melanogaster* [27], and the LD_50_ value was demonstrated to be greater than 5000 mg/kg [28]. An LD_50_ value greater than 5000 mg/kg was also confirmed with *M. spicata* [29] and *M. communis* [30] and it was greater than 2000 mg/kg for *A. herba alba* [31]. The European Medicines Agency recognizes *Origanum majorona* as a well known plant for its applications (traditional use) and recommended posology (EMA/HMPC/166517/2015).

## 3. Materials and Methods

### 3.1. Plant Material and Essential Oil Extraction

Six plants were chosen for this study. Four plants belonging to the Lamiaceae family; *Origanum compactum*, *Mentha spicata*, and *Thymus serpyllum* collected in August 2012, and *Origanum majorana* in February 2012. One myrtaceae (*Myrtus communis*) collected in August and one astraceae (*Artemisia herba-alba*) in July. All plants are from Taounate region (Morocco). A voucher specimen of each plant was deposited at the herbarium of the National Agency of Medicinal and Aromatic Plants (NAMAP), Morocco. To obtain essential oils, the fresh aerial part (leaves and stems) of each plant were subjected separately to hydrodistillation for 3 h using a Clevenger apparatus. The obtained essential oils were stored at 4 °C in dark until use. 

### 3.2. Gas Chromatography–Mass Spectrometry (GC/MS) Analysis Conditions

The analytical GC/MS system used was an Agilent GC–MSD system (Agilent Technologies 6850/5973) with helium (high purity) as the carrier gas at a constant linear velocity of 36 cm/s. The transfer, source, and quadrupole temperatures were 245 °C, 230 °C, and 150 °C, respectively, operating at 70 eV ionization energy and scanning the *m*/*z* range 50–550. The column used was an Agilent DB5 ms capillary column (30.0 m × 0.25 mm × 0.25 µm film thickness) programmed from 60 °C to 245 °C at 3 °C/min. Essential oil samples were diluted with hexane (Sigma Aldrich) (1:3000). The injected volume was 2.0 µL, in splitless mode, and the injector temperature was 250 °C. Identification of the individual components was based on: comparison with the NIST MS Search database 2012 where possible and the Adams terpene library [32].

### 3.3. Antioxidant Essays

#### 3.3.1. Determination of Inhibition Concentrations IC50 by DPPH 

The antiradical activity was evaluated by measuring the free radical scavenging activity of DPPH, using the method described Mighri et al. [33]. First, a solution of 4 mg of DPPH in 100 mL of methanol (0.004%) was prepared and then kept in the dark for 4 h. Then, a dilution series of eleven EOs of different concentrations (0.5; 1; 2; 5; 10; and 20 mg/mL) was prepared in methanol. Thus, BHT was used as a standard and prepared in methanol in a dilution series ranging from 20 to 0.002 mg/mL. Then 3 mL of solution of the samples were mixed with 3 mL of the DPPH solution. These mixtures were then kept in the dark for 30 min, then the optical density was measured at 517 nm using a JENWAY 6800 Vis-UV spectrophotometer. A total of 3 mL of methanol with 3 mL of DPPH solution (0.004%) were used as blank. The measurements were made in three repetitions. The antioxidant activity was calculated as follows:

$$\mathrm{AA}\%=\frac{{\mathrm{Abs}}_{\mathrm{control}}-{\mathrm{Abs}}_{\mathrm{Sample}}}{{\mathrm{Abs}}_{\mathrm{control}}}\times 100$$

With: AA: antioxidant activity; Abs: absorbance. The IC_50_ is calculated from the plot of serial dilutions vs the % inhibition using statistical software.

#### 3.3.2. Mixture Design and Statistical Analysis

**Figure 3:**
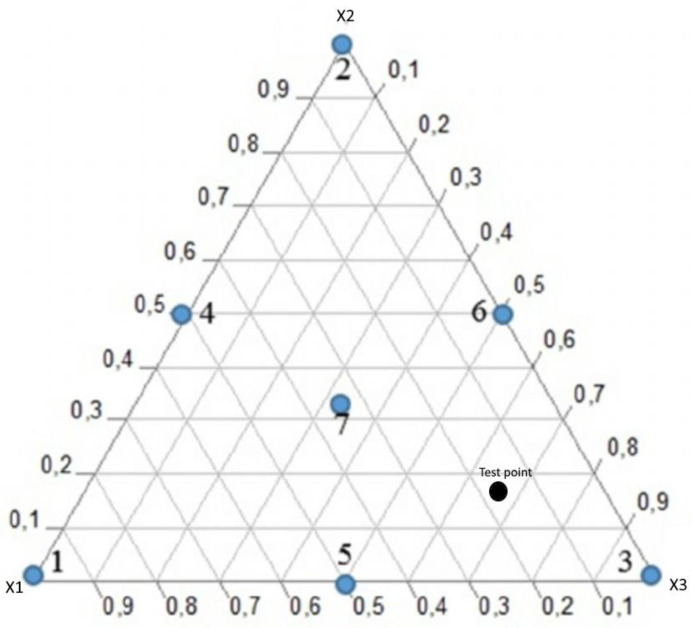
An overview of the simplex-centroid design for a three component mixture and point test.

The ternary formulation of the EOs was based on the simplex-centroid mixture design. This design was realized without constraints and with randomization. The different experiments to realize following Figure 3 surface were: The vertices of the triangle (1; 2; 3) correspond to the pure oils, the centers of the edges correspond to the mixtures half–half of two pure oils (4; 5; 6) and the center of gravity of the triangle is the mixture containing one third of each pure oil (7) (Table 8).

Three mixtures were carried out. The first mixture, M1, combined the EOs of *O. compactum*, *O. majorana*, and *T. serpyllum*; the second mixture, M2, combined Eos of *O. majorana*, *T. serpyllum*, and *M. spicata*; the third mixture, M3, associated *A. herba-alba*, *M. communis*, and *T. serpyllum* EOs (Table 2).

The formulation was carried out to obtain solutions containing 3 mg/mL. The antioxidant activity of the mixtures was tested as described below in the screening. The experimental design for each mixture is composed of 15 experiments: three replications of the central point, and two for the other experiments. 

Afterwards, the data were fitted to a special cubic polynomial model applying the least squares regression to estimate the unknown coefficients in Equation (4):

(4) $$Y={\;b}_{1}X_{1}+b_{2}X_{2}+b_{3}X_{3}+b_{12}X_{1}X_{2}+b_{13}X_{1}X_{3}+b_{23}X_{2}X_{3}+b_{123}X_{1}X_{2}X_{3}$$

where Y is the response, b_i_ the magnitude of the effect of each component X_i_, b_ij_ the magnitude of the interactive effect of two-components, and b_ijk_ is the magnitude of the interactive effect of the three components on the response. X_i_ denotes the proportions of the component (i) in the mixture. This analysis was carried out using the SAS JMP software, version 8.0.1. 

## 4. Conclusions

All Eos showed antioxidant properties with different degrees of antiradical activity. The *T. serpyllum*, *M. spicata*, and *M. communis* EOs had the highest antioxidant activity. This activity can be mainly attributed to the presence of compounds such as thymol, carvacrol, γ-terpinene, *p*-cymene, 1,8-cineol, linalool. In this paper, we also developed formulations with antioxidant activity using a mixture design method. This property has also been linked to the chemical composition. Finally, this remains a preliminary study of antioxidant interaction that must be more developed. 

## Figures and Tables

**Table 1:** Essential oils IC_50_ of DPPH.

Plants Species	IC_50_ (mg/mL)
*O. compactum*	0.49 ± 0.019
*O. majorana*	1.88 ± 0.20
*T. serpyllum*	0.07 ± 0.01
*M. spicata*	0.13 ± 0.00
*M. communis*	0.408 ± 0.006
*A. herba-alba*	3.42 ± 0.10
BHT (positive control)	0.33 ± 0.00

**Table 2:** Essential oil mixtures.

Mixture n°	EO1	EO2	EO3
1 (M1)	*O. compactum*	*O. majorana*	*T. serpyllum*
2 (M2)	*O. majorana*	*T. serpyllum*	*M. spicata*
3 (M3)	*M. communis*	*A. herba-alba*	*T. serpyllum*

**Table 3:** Original components of the design matrix and experimental responses (AA%) obtained for M1, M2, and M3 mixtures.

	EO (%) *v*/*v*			AA% of M1 *	AA% of M2 *	AA% of M3 *
Experiment	EO1	EO2	EO3			
1	50	0	50	79.55	79.55	40.93
2	100	0	0	75.13	47.3	78.5
3	33.333333	33.333333	33.333333	74.74	74.74	33.68
4	50	0	50	73.91	73.91	46.65
5	50	50	0	71.49	71.49	8.15
6	0	50	50	55.04	55.04	45.6
7	0	100	0	47.3	66.41	45.73
8	0	50	50	56.04	56.04	49.29
9	0	0	100	69.63	72.49	69.63
10	0	100	0	52.32	69.63	46.02
11	0	0	100	66.41	77.32	66.41
12	100	0	0	74.96	52.32	75.42
13	33.333333	33.333333	33.333333	73.31	73.31	36.58
14	50	50	0	74.48	74.48	5.17
15	33.333333	33.333333	33.333333	73.31	73.31	36.58

AA: antioxidant activity. * The formulation was carried out to obtain solutions containing 3 mg/mL.

**Table 4:** Analysis of variance for the different models fitted to responses.

	M1					M2					M3				
Source	ddl	SS	MS	Rapport F	*p*-Value	ddl	SS	MS	Rapport F	*p*-Value	ddl	SS	MS	Rapport F	*p*-Value
Model	6	1373.75	228.96	45.71	<0.0001	6	1369.82	228.3	35.29	<0.0001	6	6317.3	1052.9	194.97	<0.0001
Residue	8	40.08	5.01			8	51.76	6.47			8	43.2	5.4		
Total	14	1413.82				14	1421.58				14	6360.5			
R^2^	0.97					0.96					0.99				
R^2^ adjusted	0.95					0.93					0.98				

**Table 5:** Coefficients of model fitted for M1 and their level of significance determined by *p*-value.

Name	Coefficients	Estimation	*p*-Value
*O. compactum*	b_1_	75.041966	<0.0001 *
*O. majorana*	b_2_	49.81	<0.0001 *
*T. serpyllum*	b_3_	68.02228	<0.0001 *
*O. compactum/O. majorana*	b_12_	42.253951	0.0006 *
*O. compactum/T. serpyllum*	b_13_	20.784374	0.0279 *
*O. majorana/T. serpyllum*	b_23_	−13.50677	0.1197
*O. compactum/O. majorana/T. serpyllum*	b_123_	107.81547	0.0575

* *p*-value less than 0.05 is statistically significant, “1” refers to *O. compactum* coefficient, “2” referes to *O. majorana* coefficient and “3” refers to *T. serpyllum* coefficient.

**Table 6:** Coefficients of model fitted for M2 and their level of significance determined by *p*-value.

Name	Coefficient	Estimation	*p*-Value
*O. majorana*	b^1^	49.81	<0.0001 *
*T. serpyllum*	b^2^	68.02228	<0.0001 *
*M. spicata*	b^3^	74.904624	<0.0001 *
*O. majorana/T. serpyllum*	b^12^	56.293322	0.0002 *
*O. majorana/M. spicata*	b^13^	57.483617	0.0002 *
*T. serpyllum/M. spicata*	b^23^	−63.69602	<0.0001 *
*O. majorana/T. serpyllum/M. spicata*	b^123^	107.40345	0.0880

* *p*-value less than 0.05 is statistically significant, “1” refers to *O. compactum* coefficient, “2” referes to *O. majorana* coefficient and “3” refers to *T. serpyllum* coefficient.

**Table 7:** Coefficients of model fitted for M3 and their level of significance determined by *p*-value.

Name	Coefficient	Estimation	*p*-Value
*M. communis*	b_1_	76.957119	<0.0001*
*A. herba-alba*	b_2_	45.87975	<0.0001*
*T. serpyllum*	b_3_	68.02228	<0.0001*
*M. communis/A. herba-alba*	b_12_	−219.0295	<0.0001*
*M.communis/T.serpyllum*	b_13_	−114.8024	<0.0001*
*A. herba-alba/T. serpyllum*	b_23_	−38.02838	0.0015*
*M. communis/A. herba-alba/T. serpyllum*	b_123_	359.37738	0.0001*

*p-value less than 0.05 is statistically significant, “1” refers to *O. compactum* coefficient, “2” referes to *O. majorana* coefficient and “3” refers to *T. serpyllum* coefficient.

**Table 8:** Content of essential oils mixtures.

Experiment n°	EO1	EO2	EO3
1	100%	-	-
2	-	100%	-
3	-	-	100%
4	50%	50%	-
5	50%	-	50%
6	-	50%	50%
7	33.3%	33.3%	33.3%

## Data Availability

Data are available upon request.

## Supplementary Materials

The following are available online at https://www.mdpi.com/article/10.3390/plants10122817/s1, Table S1: Chemical composition of *M. communis* essential oil, Table S2: Supplementary table S2. Chemical composition of *A. herba-alba* essential oil, Table S3: Supplementary table S3. Chemical composition of *O. majorana* essential oil, Table S4: Chemical composition of *T. serpyllum* essential oil, Table S5: Chemical composition of *O. compactum* essential oil, Table S6: Chemical composition of *M. spicata* essential oil
