# Supplementary material for: Boosted Antioxidant Effect Using a Combinatory Approach with Essential Oils from Origanum compactum, Origanum majorana, Thymus serpyllum, Mentha spicata, Myrtus communis, and Artemisia herba-alba: Mixture Design Optimization
Source: Plants (Basel). 2021 Dec 20;10(12):2817. doi: 10.3390/plants10122817 (PMC8704227; doi:10.3390/plants10122817)
Supplement: Supplementary file 1 [file plants-10-02817-s001.zip › plants-1469402-supplementary.pdf]

Supplementary Table S1. Chemical composition of *M. communis* essential oil.

Compounds	Rt[a]	RI[b]	Peak area %
α-Thujene	5.28	924	0.16
α-Pinene	5.48	931	7.17
β-Pinene	6.69	977	0.13
δ-3-Carene	7.61	1009	0.15
o-Cymene	8.13	1024	1.05
Limonene	8.29	1028	8.96
**1,8-Cineole**	**8.41**	**1032**	**10.65**
cis-Linalool oxide	9.75	1070	0.27
trans-linalool oxide	10.34	1087	0.25
**Linalool**	**10.87**	**1103**	**19.77**
Terpinen-4-ol	14.13	1180	0.20
p-Cymen-8-ol	14.42	1187	0.09
cis-3-Hexenyl butyrate	14.48	1188	0.12
β-Fenchyl alcohol	14.75	1195	3.1
Estragole	14.85	1197	0.34
(*L*)-carvone	16.76	1242	0.09
Citral	17.78	1266	0.1
**Myrtenyl acetate**	**20.12**	**1321**	**33.67**
Neryl acetate	21.62	1356	0.18
Methyl eugenol	23.37	1398	0.96
trans-Caryophyllene	24.06	1415	0.22
α-Humulene	25.52	1451	0.42
4,6-diethyl-2-methoxypyrimidine	27.91	1510	1.35
Phenol, 2-(1,1-dimethylethyl)	28.56	1527	0.18
Caryophyllene oxide	30.48	1576	0.32
Humulene 1,2-epoxide	31.56	1604	0.41
**Monoterpene hydrocarbons**			17.62
**Oxygenated monoterpenes**			35.16
**Sesquiterpene hydrocarbons**			0.64
**Oxygenated sesquiterpenes**			0.73
**Others**			36.15
**Total**			90.3

a: Retention time on DB-5 capillary column in minutes.

b: Retention index

Supplementary Table S2. Chemical composition of *A. herba-alba* essential oil.

Compounds	Rt[a]	RI[b]	Peak area %
α-Pinene	5.48	931	0.10
β-Myrcene	6.96	987	0.09
p-cymene	8.13	1023	1.31
(*D*)-Limonene	8.28	1028	0.20
β-Thujene	8.35	1029	0.04
Lavender lactone	8.51	1034	0.12
γ-Terpinene	9.29	1056	0.14
3-Carene	10.84	1099	0.11
cis-4-Thujanol	12.55	1141	0.14
Fenchone	13.39	1162	0.43
Terpinen-4-ol	14.12	1179	0.68
krypton	14.37	1185	0.23
(*A*)-Terpineol	14.75	1195	1.77
**Piperitone**	**17.26**	**1254**	**85.68**
Copaene	22.28	1372	0.15
(-)-Spathulenol	30.32	1572	0.15
(+)-Spathulenol	30.32	1572	0.12
Davanone	30.44	1575	3.12
**Monoterpene hydrocarbons**			**1.99**
**Oxygenated monoterpenes**			**88.7**
**Sesquiterpene hydrocarbons**			**0.15**
**Oxygenated sesquiterpenes**			**3.38**
**Others**			**0.35**
**Total**			**94.58**

a: Retention time on DB-5 capillary column in minutes.

b: Retention index

Supplementary Table S3. Chemical composition of O. ***majorana essential oil.***

Compounds	Rt[a]	RI[b]	Peak area %
(-)-Terpinene-4-ol	14.12	1180	29.11
(*Z*)-4-Thujanol	10.93	1101	24.57
p-cymene	8.14	1023	12.64
α-Terpineol	14.74	1195	9.05
(*E*)-β-Terpineol	9.75	1069	5.45
(*Z*)-2-Menthenol	11.84	1124	2.16
β-Phellandrene	6.52	971	2.14
(*Z*)-Sabinene hydrate acetate	17.19	1252	1.71
(*E*)-Sabinene hydrate acetate	17	1248	1.48
(*E*)-*p*-Menth-2-en-1-ol	12.56	1141	1.27

Caryophyllene oxide	30.48	1576	1.08
1,8-Cineole	8.39	1031	1.05
Limonene	8.28	1028	0.98
Spathulenol	30.32	1572	0.82
ϱ-Cymene-8-ol	14.41	1186	0.59
β-Myrcene	6.95	987	0.4
Caryophyllene	24.05	1415	0.4
α-Pinene	5.48	932	0.35
β-Pinene	6.68	977	0.19
Sabinene	5.28	924	0.17
Verbenone	15.11	1203	0.11
**Monoterpene hydrocarbons**			16.87
**Oxygenated monoterpenes**			76.55
**Sesquiterpene hydrocarbons**			0.4
**Oxygenated sesquiterpenes**			1.9
**Others**			95.72

a: Retention time on DB-5 capillary column in minutes.

b: Retention index

Supplementary Table S4. Chemical composition of *T. serpyllum essential oil.*

Compounds	Rt[a]	RI[b]	Peak area %
p-Cymene	8.14	1023	36.16
γ-Terpinene	9.3	1056	18.31
Thymol	18.48	1292	17.29
Linalool	10.85	1099	4.51
Caryophyllene	24.05	1415	3.13
Thymol methyl ether	16.15	1237	3.02
α-Terpinene	7.87	1016	2.61
Limonene	8.28	1028	2.2
β-myrcene	6.95	987	1.88
Sabinene	5.28	924	1.75
α-Pinene	5.48	932	1.55
Camphene	5.92	948	0.66
β-Pinene	6.68	977	0.62
Borneol	13.78	1171	0.59
(-)-Terpinene-4-ol	14.12	1180	0.54
(-)-Camphor	12.74	1146	0.39
β-Thujene	8.35	1029	0.37
α-Phellandrene	7.53	1007	0.34
β-Farnesene	30.48	1576	0.28
Terpinolene	10.29	1084	0.25
p-Cymenene	10.48	1089	0.22
1,8-cineole	8.39	1031	0.18
α-Terpineol	14.74	1195	0.15
(+)-4-carene	9.75	1069	0.13
3-Octanone	6.82	982	0.11
(+)-3-Carene	7.6	1009	0.1
**Monoterpene hydrocarbons**			65.4
**Oxygenated monoterpenes**			28.87
**Sesquiterpene hydrocarbons**			3.533
**Oxygenated sesquiterpenes**			0
**Others**			0.09
**Total**			97.89

a: Retention time on DB-5 capillary column in minutes.

b: Retention index

Supplementary Table S5. Chemical composition of *O. compactum essential oil.*

Compounds	Rt[a]	RI[b]	Peak area %
**Carvacrol**	18.88	1301	47.85
**γ-Terpinene**	9.3	1056	17.25
**Thymol**	18.48	1292	15.75
p-Cymene	8.14	1023	8.44
α-Terpinene	7.87	1016	2.19
Caryophyllene	24.05	1415	1.44
β-Myrcene	6.95	987	1.42
Linalool	10.85	1099	1.36
Sabinene	5.28	924	1.06
α-Pinene	5.48	932	0.61
(-)-Terpinene-4-ol	14.12	1180	0.43
Limonene	8.28	1028	0.25
α-Phellandrene	7.53	1007	0.24
β-Pinene	6.68	977	0.21
β-Thujene	8.35	1029	0.21
α-Terpineol	14.74	1195	0.16
Borneol	13.78	1171	0.15
2-Carene	10.28	1084	0.12
Camphene	5.92	948	0.09
3-Octanone	6.82	982	0.09
p-Cymene	10.48	1089	0.08
Myrtenol	14.84	1197	0.08
(+)-3-Carene	7.6	1009	0.07
cis-β-Ocimene	8.84	1043	0.06
**Monoterpene hydrocarbons**			65.87
**Oxygenated monoterpenes**			32.01
**Sesquiterpene hydrocarbons**			1.44
**Oxygenated sesquiterpenes**			0
**Others**			0.09
**Total**			99.65

a: Retention time on DB-5 capillary column in minutes.

b: Retention index

Supplementary Table S6. Chemical composition of *M. spicata essential oil.*

Compounds	Rt[a]	RI[b]	Peak area %
Carvone	21.69	1358	26.00
1,8-cineole	8.41	1031	15.20
β-Myrcene	6.96	987	12.50
Limonene	8.29	1028	10.29
(*D*)-Germacrene	26.56	1476	6.86
Caryophyllene	24.06	1415	6.41
β-Pinene	6.69	977	3.03
1-Butanone	23.11	1392	2.08
α-Pinene	5.49	932	1.72
β-Phellandrene	6.53	971	1.58
(*E*)-β-Famesene	25.51	1451	1.18
γ-Elemene	27.13	1490	0.77
(*E*)-β-ocimene	8.47	1033	0.75
Pentanoic acid	16.42	1234	0.47
(-)-β-Bourbonene	22.59	1380	0.43
γ-Terpinene	9.3	1056	0.37
Limonene	14.74	1194	0.35
Resorcinol	12.19	1132	0.34
β-Elemene	22.86	1386	0.34
Bicyclo[4.4.0]dec-1-ene, 2-isopropyl-5-methyl-9-methylene-	25.79	1458	0.32
β-Methylallylbenzene	10.49	1089	0.30
α-Terpinolene	10.29	1084	0.28
2-Methylbutyl valerate	11.16	1107	0.26
(E,Z)-2,4-Hexadiene	20.11	1321	0.26
(*D*)-cadinene	28.06	1514	0.25
(+/-)-Pulegone	16.5	1236	0.24
5-Isopropenyl-2-methylcyclopent-1-enecarboxaldehyde	20.71	1335	0.24
Citronellol	16.07	1226	0.23
Isovaleric acid	11.09	1105	0.23

Terpinene-4-ol	14.12	1179	0.23
(+)-4-Carene	7.87	1016	0.23
cis-Sabinen hydrate	9.75	1069	0.22
cis-(*Z*)-α-Bisabolene epoxide	30.48	1576	0.20
α-Terpineol	13.71	1169	0.19
γ-Selinene	32.03	1617	0.19
Linalool	10.85	1099	0.18
3-Octanol	7.19	996	0.17
Cymen-8-ol	14.41	1186	0.17
n-Amyl isovalerate	10.95	1102	0.16
α-Muurolene	26.35	1471	0.15
α-ocimene	8.85	1043	0.15
β-Citronellol	18.01	1271	0.15
cis-Jasmone	22.99	1389	0.15
Copaene	22.27	1372	0.14
Butanoic Acid	16.2	1229	0.14
Spatulenol	30.31	1572	0.13
Isopiperitenone	17.8	1266	0.13
I-Calamenene	28.12	1515	0.12
1.5.5-Trimethyl-6-methylene-cyclohexene	20.43	1328	0.11
**Monoterpene hydrocarbons**			31.53
**Oxygenated monoterpenes**			43.68
**Sesquiterpene hydrocarbons**			10.22
**Oxygenated sesquiterpenes**			0.33
**Others**			10.57
**Total**			96.34

a: Retention time on DB-5 capillary column in minutes.

b: Retention index
